# A Core Outcome Set for Seamless, Standardized Evaluation of Innovative Surgical Procedures and Devices (COHESIVE): A Patient and Professional Stakeholder Consensus Study

**DOI:** 10.1097/SLA.0000000000004975

**Published:** 2023-01-10

**Authors:** Kerry N.L. Avery, Nicholas Wilson, Rhiannon Macefield, Angus McNair, Christin Hoffmann, Jane M. Blazeby, Shelley Potter

**Affiliations:** * National Institute for Health Research Bristol Biomedical Research Centre, Bristol Centre for Surgical Research, Bristol Medical School: Population Health Sciences, University of Bristol, Clifton, Bristol, UK; † Gastrointestinal Surgery, North Bristol NHS Trust, Bristol, UK; ‡ Division of Surgery, Bristol Royal Infirmary, University Hospitals Bristol NHS Foundation Trust, Bristol, UK; § Bristol Breast Care Centre, North Bristol NHS Trust, Southmead Road, Bristol, UK

**Keywords:** core outcome set, Delphi technique, device approval, operative, outcome assessment, surgical procedures

## Abstract

To develop a COS, an agreed minimum set of outcomes to measure and report in all studies evaluating the introduction and evaluation of novel surgical techniques.

**Summary of Background Data:**

Agreement on the key outcomes to measure and report for safe and efficient surgical innovation is lacking, hindering transparency and risking patient harm.

**Methods:**

(I) Generation of a list of outcome domains from published innovation-specific literature, policy/regulatory body documents, and surgeon interviews; (II) Prioritization of identified outcome domains using an international, multi-stakeholder Delphi survey; (III) Consensus meeting to agree the final COS. Participants were international stakeholders, including patients/public, surgeons, device manufacturers, regulators, trialists, methodologists, and journal editors.

**Results:**

A total of 7972 verbatim outcomes were identified, categorized into 32 domains, and formatted into survey items/questions. Four hundred ten international participants (220 professionals, 190 patients/public) completed at least one round 1 survey item, of which 153 (69.5%) professionals and 116 (61.1%) patients completed at least one round 2 item. Twelve outcomes were scored “consensus in” (“very important” by ≥70% of patients and professionals) and 20 “no consensus.” A consensus meeting, involving

context: modifications, unexpected disadvantages, device problems, technical procedure completion success, patients’ experience relating to the procedure being innovative, surgeons’/operators’ experience. Other domains relate to intended benefits, whether the overall desired effect was achieved and expected disadvantages.

**Conclusions:**

The COS is recommended for use in all studies before definitive randomized controlled trial evaluation to promote safe, transparent, and efficient surgical innovation.

Surgical innovation is essential to improving patient care.^1^ Optimal innovation is undertaken safely and transparently.^2^ Unlike the pharmaceutical industry, where rigorous regulatory pathways for new products are well-established, the introduction of novel surgical procedures is less regulated and unstandardized.

One challenge to safe and transparent innovation is consistency and transparency in the selection and reporting of relevant key outcomes. The IDEAL (Idea, Development, Exploration, Assessment, Long-term follow-up) framework describes the characteristics and recommended study designs for the stages of innovation through which new surgical procedures typically pass, describing broad outcome domains that may be measured at each stage.^3^ Recently-published IDEAL reporting guidelines have further emphasised the need for transparent reporting of appropriate technical, clinical and patient-reported outcomes, harms and unintended effects.^4^ However, consensus on which specific outcomes are essential to measure at each stage has not yet been reached, and requires a rigorous, evidence-based approach.

Inconsistency in outcome selection and reporting limits evidence synthesis and impedes efficient innovation. This may protract the introduction of promising procedures, delaying definitive evaluation in larger clinical studies or randomized controlled trials (RCT). Alternatively, it may result in ineffective or harmful procedures becoming established in routine clinical practice without a sufficient evidence base. Lack of standardized outcome reporting also provides opportunity for selective reporting that may exaggerate a procedure’s benefits or underestimate its harms, compromising patient safety.^5-8^ These issues may be addressed through developing a core outcome set (COS); an agreed standardized set of outcomes that should be measured and reported, as a minimum, in all early phase studies of a novel invasive procedure. COS can improve outcome measurement and reporting in effectiveness studies and RCTs^9,10^ and could have a similar benefit to improving the evaluation and quality and consistency of reporting of early phase surgical studies. Such a COS does not currently exist.

## Aim

To develop a COS to support standardized evaluation of all innovative invasive procedures and medical devices before definitive evaluation within an RCT.

### Methods

The COS was developed in the COHESIVE study, using consensus-based methods and in accordance with principles outlined in the core outcome measures for effectiveness trials (COMET) Handbook^11^ and Core Outcome Set-STAndards for Development guidelines.^12^ The full protocol has been published,^13^ including detailed definitions of key terminology such as “invasive procedures” and “innovation.”

The study was registered on the COMET database on November 20, 2017 (http://comet-initiative.org/Studies/Details/1055).

The study comprised 3 phases: (1) generation of a list of outcome domains; (2) outcome domain prioritization in a Delphi survey; (3) a consensus meeting to agree the COS.

### Stakeholder, Patient, and Public Involvement

Stakeholders, patients, and public members were involved throughout study planning, design, conduct, and dissemination. A steering group of 24 members, including 2 patient representatives, oversaw study conduct. A patient/public advisory group were consulted throughout.

### Phase I: Generation of a List of Outcome Domains

Multiple data sources of relevance to surgical innovation were selected to generate a long list of outcome domains to include in the Delphi survey: (i) focused literature reviews of purposively-selected case studies of innovative procedures/devices^13-16^; (ii) review of recommendations for outcome selection and reporting in NHS trust New Procedure Committee documents^13,17^; (iii) review of recommendations for outcome selection and reporting in medical device regulatory body documents,^13^ and; (iv) review of transcripts of qualitative interviews with surgeons exploring their perceptions of surgical innovation.^13,18^ Relevant outcomes/domains were extracted verbatim by at least 2 researchers independently, with discrepancies discussed with the study team.

#### Outcome Domain Conceptualization

The outcome list was used to generate a conceptual framework of outcome domains. Sections of the outcome list were categorized independently by study group members and iteratively modified through discussion. Categorization continued until no further outcome domains were identified and the framework was considered complete. The framework’s comprehensiveness was examined by mapping outcomes/outcome domains identified from a random sample of articles included in a systematic review of early phase studies of colorectal cancer surgery.^19^ Minor refinements were made where necessary, following discussion.

### Phase II: Outcome Domain Prioritization

A sequential, multi-round online Delphi survey prioritized domains with stakeholders.

#### Delphi Survey Questionnaire Development

Outcome domains identified were formatted into survey questionnaire items, written in plain English and piloted with 2 independent surgeons and 2 patient/public advisory group members to confirm comprehensibility and acceptability and refine terminology, layout, and formatting. Respondents scored the importance of including each item in the final COS on a 9-point Likert scale ranging from 1 (not important) to 9 (extremely important). Each item was scored for 4 stages of innovation, broadly consistent with the IDEAL framework^3^: (i) Early phase: first few uses of the procedure/device in humans; (ii) Development stage: procedure is evolving/being refined; (iii) Comparison stage: procedure is no longer evolving/changing (stable) and ready to be compared with standard treatments; (iv) Long-term evaluation stage: long-term outcomes of procedure. Although the scope of the COS was intended for evaluation of novel invasive procedures/devices before definitive RCT evaluation (comparison stage), this approach allowed a detailed exploration of outcome relevance across all evaluation stages. Free-text items allowed participants to propose additional outcomes, which were included in subsequent round(s) if the outcome was considered to be new and was proposed by at least 2 participants.

#### Sampling and Recruitment

Key stakeholders relevant to developing the COS and participating in the Delphi survey included surgeons, funding bodies, device industries, and small and medium-sized enterprises, trialists, methodologists, journal editors, regulators, and patient representatives. Patient representatives were approached through a range of partner organisations and purposively sampled based on sex, age, geographical region, and surgical procedure to enable inclusion of a diverse range of demographics and experiences.

Professional participants were purposively sampled to include international representation and a range of professional backgrounds (eg, surgeons, speciality professional associations, industry collaborators, device manufacturers). Identification and sampling of professionals was informed by: (i) expert knowledge of study team and study steering group members and their colleagues; (ii) contact lists held by the Centre for Surgical Research (University of Bristol) of attendees at surgical-related academic events (eg, attendees at relevant conferences/workshops); (iii) review of public websites and other public resources; and (iv) specialty professional associations directly circulating the survey to their membership via email.

Additional patient and professional participants were recruited by advertising on social platforms (eg, twitter: @CohesiveStudy) and/or participants opting-in to complete the Delphi survey via the open-access COHESIVE study website.^20^

#### Delphi Survey Rounds

Participants completed 2 sequential survey rounds (1: July–September 2019; 2: October–November 2019). Survey questionnaires were administered online, facilitated by secure Research Electronic Data Capture (REDCap) electronic data capture soft-ware,^21^ and in accordance with the Checklist for Reporting Results of Internet E-Surveys (CHERRIES) guidelines.^22^ A paper-based survey was available for postal administration, if requested. All round 1 respondents were invited to complete round 2. All items were retained between rounds 1 and 2, providing opportunity for participants to re-score items taking into consideration feedback from their own and the other stakeholder group.^11,23,24^ Anonymized feedback from round 1, including the respondent’s own score and the median score from each stakeholder group (patients and professionals, displayed separately) for each item was included next to each item. This enabled each stakeholder group to see the other’s results before re-scoring each item, to encourage prioritization. In round 2, participants re-scored each item’s importance. It was agreed a priori that a third round may be considered if sufficient consensus to proceed to the consensus meeting was not reached after round 2.

A definition of consensus was outlined a priori (Table 1). Following discussion within the study steering group a post hoc decision was made to use a stricter definition of consensus after round 2 due to the high proportion of items scored as “extremely important,” as it was felt unlikely that a third survey round would result in any further prioritization. Data was analyzed in Stata.^25^ Only items/domains relevant to the “early phase” or “development stage” of innovation were taken to the consensus meeting.

**Table 1:** Definition of Consensus

	Original (Rounds 1 and 2)		Revised (Round 2 Only)		Consensus Meeting	
Category	Definition	Action	Definition	Action	Definition	Action
Consensus in	Scored as very important **(7–9**)by ≥70% and not important (1–3) by <15% of either patients or professionals	Domain retained for next survey round/consensus meeting	Scored as very important **(8–9)** by ≥70% and not important (1–3) by <15% of patients and professionals	Domain retained for ratification at consensus meeting	Same as round 2	Domain included in final COS, following ratification*
Consensus out	Scored as not important (1–3) by ≥70% and very important (7–9) by <15% of either patients or professionals or both	Domain discarded after round 2 (to be ratified at consensus meeting)	Same as original	Same as original	Same as original	Domain not included in final COS, following ratification
No consensus	Neither criteria above are met	Domain retained for next survey round/consensus meeting	Neither above criteria are met	Domain sub-categorized:		
				(i) scored “very important” (median 8–9) by ≥70% of either patients or professionals	Same as round 2	Iterative rounds of discussion and voting until consensus achieved
				(ii) scored “very important” (median 8–9) by 50%–70% of either patients or professionals	Same as round 2	Iterative rounds of discussion and voting until consensus achieved
				(iii) scored “very important” (median 8–9) by ≤50% of both patients and professionals	Same as round 2	Domain excluded from final COS, following ratification

* Either as an individual or grouped with another domain(s). COS indicates core outcome set.

#### Attrition

Participant attrition between rounds was monitored. Automatic reminder emails were sent to participants who had started but not completed the survey.

### Phase III: Consensus Meeting

The consensus meeting was held in Bristol, UK, in February 2020. Participants were purposively sampled to include representation from key stakeholder groups. The meeting was facilitated by an independent chair who had not been involved in the study’s design/conduct. The chair encouraged discussion and ensured that all participants were able to freely express their views.

A summary of the survey results was presented. Participants were asked to ratify the inclusion or exclusion of outcome domains categorized as “consensus in” or “consensus out” following Phase II. Discussion and voting were undertaken for outcome domains/items where objections were raised and for items where no consensus was reached during the Delphi survey. Participants were asked to vote items “In” or “Out” of the COS. Items voted “In” by ≥70% of participants were included in the final COS. Decisions to group items were made following moderated discussion and further voting. All other items were discarded. Voting was conducted anonymously, using electronic polling software (TurningPoint). Participants unable to attend in person were offered an option to listen (though not contribute) to the discussion via teleconference and vote remotely. The consensus meeting concluded with asking participants to vote to ratify the final COS.

### Sample Size

In the absence of formal guidance for sample size calculations for COS development, consideration is given to achieving representation from all key stakeholder groups.^11^ A target sample of 150 professional and 50 patient survey participants and 20–25 consensus meeting participants from all stakeholder groups was agreed, in line with similar research.^11^ A 3:1 ratio of professionals to patients was considered appropriate to sample multiple sub-groups of professional stakeholders.

### Ethics and Dissemination

Ethical approval was granted by North East - Newcastle and North Tyneside 1 Health Research Authority Research Ethics Committee (18/NE/0378). Written informed consent was obtained from participating patients separately for the Delphi survey and consensus meeting. Written consent was obtained from professional participants before the consensus meeting.

## Results

### Phase I: Generation of a List of Outcome Domains

A final long list comprising 7972 verbatim outcomes was identified from all data sources included in Phase I.^13–16,18^ Following de-duplication and categorization, 32 outcome domains were included in the long list/conceptual framework. Of these, 22 were considered conceptually specific to evaluating innovation and 10 conceptually similar to outcomes measured in effectiveness studies. Some 2073 verbatim outcomes extracted from 51 studies identified from the systematic review of early phase studies of colorectal cancer surgery were successfully mapped to confirm the comprehensiveness of the conceptual framework, with some minor refinements.^19^

### Phase II: Outcome Domain Prioritization

The 32 outcome domains were formatted into Delphi survey questionnaire items, each with 4 components to distinguish the 4 prespecified stages of evaluation.

#### Round 1

Some 410 participants, including 220 professionals and 190patients/public, completed at least 1 questionnaire item (Supplemental Table 1, http://links.lww.com/SLA/D152). Most (n = 168, 76%) professionals were male and aged 30–60 years (n = 188, 85%). Around half (108, 49%) were from the UK, with the remainder from Europe (45, 21%), the Americas (n = 34, 16%), Asia (n = 17, 8%), Australasia (n = 8), Africa (n = 6), and the Middle East (n = 2). Over half of professionals identified as consultant surgeons/attending physicians (n = 131, 60%) or trainee/resident surgeons (n = 36, 16%). One quarter (n = 58, 26%) of professionals identified as researchers/academics/trialists/methodologists. Professional participants also included journal editors (n = 10, 5%), industry representatives (n = 6), regulatory representatives (n = 5), allied healthcare professionals (n = 2), and an anesthetist (n = 1). Professionals represented all major surgical specialties, including general (n = 38, 17%), neurological (n = 37, 17%), colorectal (n = 35, 16%), orthopaedic (n = 29, 13%), and emergency (n = 21, 10%) surgery. Over half of patients were female (n = 115, 61%) and most were aged 50 and over (n = 148, 78%). Most (n = 180, 95%) were from the UK, with the remainder from Europe, Asia, North America, Australasia, and the Middle East. All patients had previous experience of undergoing surgery of varying types and severity. Round 1 outcome domain scores are shown in Supplemental Table 2, http://links.lww.com/SLA/D153. No new items were proposed by more than 1 participant.

### Round 2

Some 153 (69.5%) professionals and 116 (61.1%) patients who scored at least one round 1 item participated in round 2 (and completed at least 1 round 2 item). Participant demographic characteristics were similar between rounds (Supplemental Table 1, http://links.lww.com/SLA/D152). Twelve of the 32 outcome domains were scored “consensus in” (“very important” by ≥70% of patients and professionals) and were carried forward to the consensus meeting to ratify their inclusion in the final COS (Supplemental Table 2, http://links.lww.com/SLA/D153). Of the remaining 20 domains, 7 were scored “very important” by ≥70% of only either patients or professionals and nine “very important” by 50%–70% of either patients or professionals, and were carried forward for further discussion and voting at the consensus meeting. The remaining 4 domains were scored “very important” by ≤50% of either patients and professionals and were carried forward to the consensus meeting to ratify their exclusion.

**Table 2:** Final Core Outcome Set

	Domain Discussed During Consensus Meeting*	Domain Decided at Consensus Meeting* (n = 10)	Final COS Domain (n = 8)
9	Anticipated advantages during	Intended benefits before, during or after the procedure	†Intended benefit(s) of the procedure, including (i) before, (ii) during or (iii) after the procedure (eg, fewer tests needed before surgery, less operative time, better recovery)
10	Unanticipated advantages during		
11	Anticipated short-term advantages following		
12	Unanticipated short-term advantages following		
13	Anticipated long-term advantages following		
14	Unanticipated long-term advantages following		
5	Whether any accompanying intervention of the innovative procedure required modifications	Whether any accompanying intervention of the innovative procedure required modifications	‡Modifications to the (i) procedure, (ii) concomitant interventions or (iii) which patients were offered the procedure during the study. NB: excludes abandoning or changing to another procedure at any point (eg, laparoscopic approach converted to open)
30	Whether changes were made to which patients were offered the innovative procedure	Whether changes were made to which patients were offered the innovative procedure	
2	Whether the planned innovative procedure was abandoned or changed	Whether the planned innovative procedure was abandoned or changed	‡Procedure completion success, either with/without modifications
1	Whether all the technical steps of the innovative procedure were completed as planned	Technical steps completed as planned, either with/without	
3	Whether any individual technical steps of the planned innovative procedure required modifications	modifications, details of what and why	
4	Details of any modifications		
7	DEVICES: Whether the new device mechanically/technically did not function as intended	DEVICES: Whether the new device mechanically/technically did not function as intended	‡Problems with the device working (eg, new stapler misfired), if applicable
15	Anticipated disadvantages during	Expected and unexpected disadvantages before, during and after the procedure	†(a) Expected disadvantages, including (i) before, (ii) during or (iii) after the procedure
16	Unanticipated disadvantages during		
17	Anticipated short-term disadvantages after		(eg, more tests needed before surgery, longer operative time, more patients required intensive care)
18	Unanticipated short-term disadvantages after		
19	Anticipated long-term disadvantages		
20	Unanticipated long-term disadvantages		‡(b) Unexpected disadvantages, including (i) before, (ii) during or (iii) after the procedure (eg, unexpected instrument clashing, inadvertent injury to nearby tissue and/or organs)
6	Whether the innovative procedure was completed (as planned or with modifications) and the overall desired effect of the procedure was achieved	Whether the overall desired effect of the procedure was achieved	Overall desired effect (overall aim) of the procedure/device achieved (eg, tumor successfully excised)
8	Operators’† perceptions/experiences	Surgeons’/operators’ perceptions/experiences of performing the innovative procedure (before, during, and after)	‡Surgeons’/operators’ emotional, psychological, or physical experience of the procedure (eg, ergonomic comfort during the operation)
27	Patients’ physical experiences during	Patients’ experience (multidomain) before, during, and after the procedure	‡Patients’ emotional, psychological, or physical experience relating to the procedure being innovative (eg, anxiety because of the procedure being new)
28	Patients’ psychological or emotional experiences		
29	Patients’ experiences following		

* Abbreviated description.

† Shared with effectiveness studies

‡ Innovation-specific.

COS indicates core outcome set.

### Phase III: Consensus Meeting

Ten patient/public representatives attended the consensus meeting (7 male, 3 female). Nine were from the UK and 1 from Europe. All had experience of undergoing at least 1 invasive procedure. Nineteen professionals (10 female, 9 male) attended, including 10 surgeons or attending physicians, 6 researchers/academics/trialists/methodologists, 2 members of hospital trust or National Institute for Health Research clinical research organizations, and 1 industry representative. Seventeen professionals were from the UK, 1 from the USA, and 1 from Europe.

Participants agreed to include 24 of the 32 Delphi survey outcome domains in the final COS (Supplemental Table 3, http://links.lww.com/SLA/D154). Of these 24 included domains, 12 were scored “consensus in” during the Delphi survey and 12 were included following additional discussion and voting during the consensus meeting. Eight domains were excluded.

During consensus meeting discussion, participants proposed collapsing 18 items into 4 broader outcome domains due to similarities and overlap in concepts across the 24 included outcome domains. These related to intended benefits of the procedure (6 items), disadvantages of the procedure (6 items), completion of planned technical steps either with/without modifications (3 items), and patients’ experiences (3 items). This resulted in a proposed COS comprising 10 items. All but 1 participant endorsed the proposed final COS at the end of the meeting (96% agreement). Participants agreed, however, that the terminology of some domains would need refinement by the study team after the meeting for clarity and consistency. Refinement resulted in a final COS comprising 8 domains, of which 6 are specific to the context of surgical innovation (Table 2).

## Discussion

The COHESIVE study has developed a COS to support the introduction and standardized evaluation and reporting of innovative invasive procedures and medical devices. Multiple data sources with specific relevance to innovation identified a comprehensive list of outcomes to inform a conceptual framework, including innovation-specific outcome domains. Consensus methods, involving key stakeholders in a large international survey and consensus meeting, then prioritized items for COS inclusion. The final COS comprises 8 outcome domains to measure and report and is recommended for use in all early-phase studies of innovative surgical procedures to optimize learning, minimize risk and inform full evaluation in later phase studies. Ultimately, this will protect patients, surgeons, and healthcare providers.

Standard COS that specify the outcomes that should be measured and reported, as a minimum, in all effectiveness trials of specific conditions or interventions focus on traditional clinical outcomes, such as complications and quality of life, of specific relevance to that area.^11^ The COS developed here is intentionally generic to be applicable to the full breadth of surgical innovation. This will enable consistent, rigorous evaluation of key outcomes from the earliest stages of evaluation. An independent safety review of medical devices has recently highlighted substantial flaws with current mechanisms for spotting trends in harm outcomes.^26^ The IDEAL framework describes broad outcome domains to measure that may vary across stages of evaluation, including technical achievement, disasters and dramatic successes (stage 1: Idea), technical and procedural success (2a: Development), and clinical outcomes (2b: Exploration).^3^ Regulatory guidance for introducing new devices from the US Food and Drug Administration and UK Medicines and Healthcare products Regulatory Agency focus primarily on evaluation of safety and efficacy,^27-29^ overlooking outcomes that this study shows are important to evaluating the process of innovation. The COS will facilitate surgeon innovators, methodologists, and device manufacturers in the practical application of the IDEAL framework by clarifying the essential outcomes to measure throughout the innovation life cycle.^4^ Although the COS includes some outcome domains characteristic of effectiveness studies (eg, intended benefit of the procedure, expected disadvantages), most are specific to innovation. These innovation-specific domains, including technical procedure success, modifications, and surgeon/operator experience, may not traditionally be recognized as outcomes but their measurement is key to driving safe and efficient innovation. Consistently measuring and reporting these outcomes may streamline innovation; enabling rapid identification of promising interventions for definitive RCT evaluation while allowing ineffective or potentially harmful interventions to be confidently abandoned before becoming established.

The COS was developed in accordance with the principles outlined by the COMET Initiative,^11^ though standard methods for identifying potential outcomes were modified to include data sources specifically relevant to innovation. Rigorous consensus methods, including a large survey of a diverse group of patients and professionals, ensured that the chosen outcomes are relevant and meaningful to key stakeholders. An international survey enabled the views of stakeholders with experience of different global healthcare and research systems to be included. Such inclusive stakeholder engagement is vital if the COS is to be adopted and used in practice.

Various COS development methods are available.^11^ Alternative data sources may have identified outcomes not included in the long list. Although frameworks exist to structure the categorization of outcomes into domains, these reflect conceptualizations relevant predominantly to the context of effectiveness trials or particular outcomes.^30^ It was considered important to derive a conceptual framework to categorize outcomes from data sources that were selected for their specific relevance to surgical innovation. This involved outcome categorization by multiple study group members and iterative modifications following group discussion. Comprehensiveness of the long list and suitability of the conceptual framework was assessed by mapping outcomes onto those from an independent systematic review of early phase surgical studies.^19^ Survey participants could also propose the inclusion of additional outcomes, though did not identify any new outcomes. Although sampling was designed to include international representation from a wide range of key stakeholder groups, around two-thirds of survey participants were from the UK and Europe. This may have influenced item prioritization. Regulatory processes, for example, vary internationally and it is possible that including more international regulatory representatives may have altered the findings, though this is considered unlikely. Although most patients and professionals completed both survey rounds, the survey was long and this may have increased attrition, introducing bias. A post-hoc decision to use stricter consensus definitions after round 2 was made due to the high proportion of items still scored as extremely important. This may have influenced discussion and voting during the consensus meeting. Although a third survey round could have been held, this was considered unlikely to encourage any further consensus on prioritization.

Use of a COS does not mean that outcomes in a specific study should be restricted to the COS,^11^ and we recommend additional outcomes be collected where relevant. Use of the COS is intended to complement reporting guidelines developed for authors to report studies accurately and comprehensively. For example, stage-specific checklists have recently been published to improve standards of reporting in publications of IDEAL format studies.^4^ These checklists recommend that authors declare important contextual information that may be omitted, such as sources of funding and conflicts of interest. A COS specifies what outcomes should be measured. Important next steps to improve the quality and consistency of evaluation of surgical innovation is to reach consensus on how the outcomes should be measured^31^ and to evaluate COS uptake in future evaluations. Development of a core measurement set, a set of instruments to measure the COS domains,^31^ including identifying a measure of surgeons’ experience, is underway. This involves mapping the COHESIVE COS to COS developed for effectiveness trials to identify outcomes of relevance throughout the innovation lifecycle. In parallel, work with key stakeholders has commenced to co-create a real-time reporting platform to optimize the utility and effective incremental sharing of surgical innovation and outcome data. Future work will consider implementation of the COS in surgical registries and its application to governance processes. Previous research has, for example, explored the benefits of novel methods to improve quality and safety processes for surgical innovation without impeding efficiency^32^ and optimal ethical regulation,^33^ and the COS is considered complementary to this work. Engagement with the surgical community is essential to ensure uptake of the COS and implementation of real-time outcome sharing, and this work is ongoing. If successful, these measures will promote safe, transparent, and efficient introduction and evaluation of surgical innovation to benefit patients and the wider healthcare community.

## Supplementary Material
