# Supplementary material for: A Core Outcome Set for Seamless, Standardized Evaluation of Innovative Surgical Procedures and Devices (COHESIVE): A Patient and Professional Stakeholder Consensus Study
Source: Ann Surg. 2023 Jan 10;277(2):238–45. doi: 10.1097/SLA.0000000000004975 (PMC9831031; doi:10.1097/SLA.0000000000004975)
Supplement: Supplementary file 1 [file sla-277-0238-s001.docx]

**Supplemental Table 1. Demographics of Delphi survey participants^a^**

|  |  | **Professionals** | | | | **Patients** | | | |
| --- | --- | --- | --- | --- | --- | --- | --- | --- | --- |
|  |  | **Round 1** | | **Round 2** | | **Round 1** | | **Round 2** | |
|  | | **n=220** | **%** | **n=153** | **%** | **n=190** | **%** | **n=116** | **%** |
| **Age** | |  |  |  |  |  |  |  |  |
|  | <30 | 10 | 4.5 | 10 | 6.5 | 5 | 2.6 | 3 | 2.6 |
|  | 30-39 | 64 | 29.1 | 47 | 30.7 | 9 | 4.7 | 6 | 5.2 |
|  | 40-49 | 68 | 30.9 | 52 | 34.0 | 24 | 12.6 | 9 | 7.8 |
|  | 50-59 | 56 | 25.5 | 31 | 20.3 | 38 | 20.0 | 21 | 18.1 |
|  | 60-69 | 19 | 8.6 | 12 | 7.8 | 64 | 33.7 | 49 | 42.2 |
|  | 70+ | 1 | 0.5 | 1 | 0.7 | 46 | 24.2 | 26 | 22.4 |
|  | Prefer not to say/not known | 2 | 1.0 | 0 | 0.0 | 4 | 2.2 | 2 | 1.8 |
| **Gender** | |  |  |  |  |  |  |  |  |
|  | Male | 168 | 76.4 | 114 | 74.5 | 69 | 36.3 | 43 | 37.1 |
|  | Female | 49 | 22.3 | 37 | 24.2 | 115 | 60.5 | 69 | 59.5 |
|  | Prefer not to say/not known | 3 | 1.4 | 2 | 1.3 | 6 | 3.2 | 4 | 3.4 |
| **Country/region** | |  |  |  |  |  |  |  |  |
|  | United Kingdom | 108 | 49.1 | 71 | 46.4 | 180 | 95.2 | 107 | 92.2 |
|  | Europe (Mainland) | 42 | 19.1 | 34 | 22.2 | 3 | 1.6 | 2 | 1.7 |
|  | The Americas | 34 | 15.5 | 22 | 14.4 | 1 | 0.5 | 1 | 0.9 |
|  | Asia | 17 | 7.7 | 12 | 7.8 | 2 | 1.1 | 0 | 0 |
|  | Australasia | 8 | 3.6 | 5 | 3.3 | 2 | 1.1 | 1 | 0.9 |
|  | Africa | 6 | 2.7 | 7 | 4.6 | 0 | 0 | 0 | 0 |
|  | Republic of Ireland | 3 | 1.4 | 1 | 0.7 | 1 | 0.5 | 1 | 0.9 |
|  | Middle East | 2 | 0.9 | 1 | 0.7 | 1 | 1 | 0 | 0 |
| **Specialty^b^** | |  |  |  |  |  |  |  |  |
|  | Consultant surgeon/attending physician | 131 | 59.5 | 88 | 57.5 |  |  |  |  |
|  | Researcher/Academic/Trialist/Methodologist | 58 | 26.4 | 42 | 27.5 |  |  |  |  |
|  | Trainee/Resident surgeon | 36 | 16.4 | 29 | 19 |  |  |  |  |
|  | Journal editor | 10 | 4.5 | 7 | 4.6 |  |  |  |  |
|  | Small/medium enterprise representative | 6 | 2.7 | 5 | 3.3 |  |  |  |  |
|  | Regulatory related agency/field representative | 5 | 2.3 | 2 | 1.3 |  |  |  |  |
|  | Allied health care professional | 2 | 0.9 | 2 | 1.3 |  |  |  |  |
|  | Other | 8 | 3.7 | 4 | 2.7 |  |  |  |  |
| **Years as consultant/attending physician** | |  |  |  |  |  |  |  |  |
|  | <5 | 24 | 18.3 | 19 | 21.6 |  |  |  |  |
|  | 5-10 | 32 | 24.4 | 22 | 25.0 |  |  |  |  |
|  | >10 | 75 | 57.3 | 47 | 53.4 |  |  |  |  |
| **Surgical specialty^b^** | |  |  |  |  |  |  |  |  |
|  | General^c^ | 163 | - | 117 | - |  |  |  |  |
|  | Neurosurgery | 37 | 16.8 | 28 | 31.8 |  |  |  |  |
|  | Orthopedic | 29 | 13.2 | 20 | 22.7 |  |  |  |  |
|  | Plastic and reconstructive | 7 | 3.2 | 4 | 4.5 |  |  |  |  |
|  | Spine (orthopaedic) | 7 | 3.2 | 6 | 6.8 |  |  |  |  |
|  | Vascular | 7 | 3.2 | 4 | 4.5 |  |  |  |  |
|  | Pediatric | 6 | 2.7 | 5 | 5.7 |  |  |  |  |
|  | Otorhinolaryngological | 5 | 2.3 | 2 | 2.3 |  |  |  |  |
|  | Oncology | 3 | 1.4 | 2 | 2.3 |  |  |  |  |
|  | Oral and maxillofacial | 2 | 0.9 | 2 | 2.3 |  |  |  |  |
|  | Thoracic | 2 | 0.9 | 1 | 1.1 |  |  |  |  |
|  | Urological | 2 | 0.9 | 2 | 2.3 |  |  |  |  |
|  | Cardiac | 1 | 0.5 | 0 | 0 |  |  |  |  |
|  | Gynecology | 1 | 0.5 | 1 | 1.1 |  |  |  |  |
|  | Ophthalmic | 1 | 0.5 | 2 | 2.3 |  |  |  |  |
|  | Other | 1 | 0.5 | 1 | 1.1 |  |  |  |  |
| **Elective or emergency surgery^b^** | |  |  |  |  |  |  |  |  |
|  | Elective/Planned |  |  |  |  | 167 | 87.9 | 99 | 85.3 |
|  | Emergency/Unplanned |  |  |  |  | 23 | 12.1 | 33 | 28.4 |
| **Surgery required overnight hospital stay** | |  |  |  |  |  |  |  |  |
|  | Yes |  |  |  |  | 164 | 86.3 | 97 | 83.6 |
|  | No |  |  |  |  | 24 | 12.6 | 16 | 13.8 |
|  | Not known |  |  |  |  | 2 | 1.1 | 3 | 2.6 |
| **Type of surgery^b^** | |  |  |  |  |  |  |  |  |
|  | General^d^ |  |  |  |  | 199 | - | 114 | - |
|  | Orthopedic |  |  |  |  | 29 | 15.3 | 20 | 17.2 |
|  | Cardiac |  |  |  |  | 22 | 11.6 | 16 | 13.8 |
|  | Ophthalmic |  |  |  |  | 14 | 7.4 | 11 | 9.5 |
|  | Cosmetic |  |  |  |  | 1 | 0.5 | 1 | 0.9 |
|  | Other |  |  |  |  | 42 | 22.1 | 20 | 17.2 |
| **Medical device inserted into body** | |  |  |  |  |  |  |  |  |
|  | Yes |  |  |  |  | 74 | 38.9 | 69 | 59.5 |
|  | No |  |  |  |  | 116 | 61.1 | 47 | 40.5 |
| **Approximate number of surgeries** | |  |  |  |  |  |  |  |  |
|  | 1 |  |  |  |  | 22 | 11.6 | 13 | 11.4 |
|  | 2-5 |  |  |  |  | 115 | 60.4 | 72 | 63.2 |
|  | 6-9 |  |  |  |  | 31 | 16.3 | 17 | 14.8 |
|  | ≥10 |  |  |  |  | 20 | 10.5 | 12 | 10.5 |
| **When was your last surgery?** | |  |  |  |  |  |  |  |  |
|  | ≥5 years ago |  |  |  |  | 71 | 37.4 | 44 | 38.3 |
|  | <5 years ago |  |  |  |  | 119 | 62.6 | 71 | 61.7 |
| **What is your highest education level?** | |  |  |  |  |  |  |  |  |
|  | Secondary education/High School |  |  |  |  | 37 | 19.4 | 18 | 15.7 |
|  | Post-secondary education |  |  |  |  | 35 | 18.3 | 20 | 17.4 |
|  | Bachelor or equivalent |  |  |  |  | 57 | 29.8 | 36 | 31.3 |
|  | Master or equivalent |  |  |  |  | 47 | 24.6 | 30 | 26.1 |
|  | Doctoral or equivalent |  |  |  |  | 14 | 7.3 | 11 | 9.6 |

^a^ participants scoring at least at least 1 COS item

^b^ participants were able to select more than one option

^c^ including colorectal, emergency, trauma, esophagogastric, hepatobiliary, breast, bariatric, endocrine, abdominal wall, transplant

^d^ including bowel, cancer, appendix, hernia, breast
