# Supplementary material for: A Core Outcome Set for Seamless, Standardized Evaluation of Innovative Surgical Procedures and Devices (COHESIVE): A Patient and Professional Stakeholder Consensus Study
Source: Ann Surg. 2023 Jan 10;277(2):238–45. doi: 10.1097/SLA.0000000000004975 (PMC9831031; doi:10.1097/SLA.0000000000004975)
Supplement: Supplementary file 2 [file sla-277-0238-s002.docx]

**Supplemental Table 2. Scoring of outcome domains in the Delphi survey**

|  |  | **Round 1 score (n=410^a^)** | | | | **Round 2 score (n=269^a^)** | | | |  |
| --- | --- | --- | --- | --- | --- | --- | --- | --- | --- | --- |
|  |  | **Professionals (n=220)** | | **Patients (n=190)** | | **Professionals (n=153)** | | **Patients (n=116)** | |  |
| **Outcome/outcome domain** | **Phase/Stage** | **1-3 (%)** | **7-9 (%)** | **1-3 (%)** | **7-9 (%)** | **1-3 (%)** | **7-9 (%)** | **1-3 (%)** | **7-9 (%)** | **Consensus decision after round 2**^b^ |
| 1: Whether all the technical steps of the innovative procedure were completed as planned (e.g. number, rate or proportion of patients where this happened) | Early | 6.4 | 71.4 | 8.4 | 72.3 | 0.7 | 75.8 | 4.3 | 65.5 | No consensus^c^ |
|  | Development | 2.7 | 74.1 | 3.2 | 76.8 | 0.7 | 75.2 | 4.4 | 69.6 | No consensus^c^ |
| 2: Whether the planned innovative procedure was abandoned or changed to an alternative procedure (e.g. keyhole (small cuts) changing to open (big cuts)) | Early | 5.9 | 74.9 | 7.5 | 75.5 | 1.3 | 79.7 | 4.3 | 68.4 | No consensus^c^ |
|  | Development | 1.8 | 79.0 | 2.7 | 80.3 | 1.3 | 82.4 | 2.6 | 73.5 | In |
| 3: Whether any individual technical steps of the planned innovative procedure required modifications (e.g. number, rate, or proportion of patients where this happened) | Early | 5.1 | 71.3 | 3.8 | 72.8 | 0.0 | 75.0 | 2.7 | 65.5 | No consensus^c^ |
|  | Development | 2.3 | 79.2 | 1.3 | 77.4 | 0.0 | 80.9 | 1.8 | 71.7 | In |
| 4: Details of any modifications to the planned innovative procedure (i.e. what the modification was, why it was made) | Early | 3.7 | 73.8 | 7.0 | 79.6 | 0.7 | 82.8 | 3.6 | 73.9 | In |
|  | Development | 1.4 | 79.4 | 1.9 | 82.3 | 0.7 | 85.4 | 0.9 | 77.5 | In |
| 5: Whether any accompanying (concomitant) intervention of the innovative procedure required modifications (e.g. antibiotics, drains, dressings or analgesia) | Early | 6.1 | 69.2 | 6.5 | 80.5 | 0.7 | 74.2 | 1.8 | 73.0 | In |
|  | Development | 3.7 | 76.2 | 0.7 | 81.9 | 0.7 | 77.5 | 0.9 | 73.9 | In |
| 6: Whether the innovative procedure was completed (as planned or with modifications) and the overall desired effect of the procedure was achieved (e.g. tumour completely excised, biopsy of enough quality for diagnosis) | Early | 1.4 | 74.4 | 4.0 | 82.9 | 0.0 | 79.5 | 1.9 | 83.2 | In |
|  | Development | 1.4 | 79.0 | 0.7 | 86.0 | 0.0 | 84.8 | 0.9 | 83.2 | In |
| 7: FOR DEVICES: Whether the new device mechanically/technically did not function as intended, including details of what did not function (e.g. pacemaker battery did not work as planned) | Early | 2.4 | 87.0 | 2.6 | 86.2 | 1.3 | 90.7 | 1.8 | 82.7 | In |
|  | Development | 1.0 | 90.4 | 1.3 | 90.8 | 0.7 | 92.1 | 0.9 | 84.6 | In |
| 8: Operators'* perceptions/experiences of performing the innovative procedure  (e.g. ease of use for surgeon)  **this term applies to anyone performing the procedure* | Early | 7.2 | 63.5 | 4.6 | 80.9 | 2.7 | 64.9 | 1.8 | 70.9 | No consensus^c^ |
|  | Development | 3.9 | 72.6 | 2.0 | 82.9 | 1.3 | 70.2 | 0.9 | 73.6 | In |
| 9: Anticipated advantages during the innovative procedure (e.g. faster, less damage to parts of the body) | Early | 6.3 | 63.3 | 4.0 | 74.5 | 0.7 | 49.7 | 1.8 | 60.6 | No consensus^d^ |
|  | Development | 3.9 | 71.5 | 0.7 | 83.2 | 0.7 | 53.6 | 0.9 | 63.3 | No consensus^d^ |
| 10: Unanticipated advantages during the innovative procedure | Early | 4.4 | 66.7 | 4.7 | 79.2 | 2.0 | 50.3 | 2.8 | 60.6 | No consensus^d^ |
|  | Development | 2.9 | 73.0 | 3.4 | 82.6 | 1.3 | 55.0 | 1.8 | 63.3 | No consensus^d^ |
| 11: Anticipated short-term advantages following the innovative procedure (e.g. faster post-operative recovery, less post-operative pain, fewer post-operative problems) | Early | 3.9 | 67.0 | 2.8 | 79.9 | 1.3 | 52.0 | 2.8 | 63.6 | No consensus^d^ |
|  | Development | 2.4 | 73.3 | 0.0 | 84.7 | 0.7 | 62.7 | 0.9 | 66.4 | No consensus^d^ |
| 12: Unanticipated short-term advantages following the innovative procedure | Early | 3.9 | 65.5 | 4.2 | 77.1 | 2.0 | 48.0 | 3.7 | 56.1 | No consensus^d^ |
|  | Development | 3.4 | 75.2 | 1.4 | 81.3 | 2.0 | 52.7 | 2.8 | 58.9 | No consensus^d^ |
| 13: Anticipated long-term advantages following the innovative procedure | Early | 10.7 | 49.0 | 4.2 | 78.5 | 3.4 | 28.9 | 2.8 | 53.3 | No consensus^d^ |
|  | Development | 8.3 | 58.7 | 2.1 | 83.3 | 3.4 | 36.2 | 0.9 | 57.0 | No consensus^d^ |
| 14: Unanticipated long-term advantages following the innovative procedure | Early | 12.6 | 48.1 | 8.3 | 76.4 | 4.0 | 28.2 | 4.7 | 56.1 | No consensus^d^ |
|  | Development | 9.7 | 57.3 | 4.2 | 79.2 | 4.0 | 34.2 | 1.9 | 54.2 | No consensus^d^ |
| 15: Anticipated disadvantages (including complications) during the innovative procedure (e.g. bleeding, damage to other organs) | Early | 2.4 | 82.0 | 1.4 | 92.9 | 0.0 | 83.1 | 1.9 | 80.2 | In |
|  | Development | 1.5 | 87.8 | 0.0 | 94.3 | 0.0 | 84.5 | 0.9 | 84.9 | In |
| 16: Unanticipated disadvantages (including complications) during the innovative procedure | Early | 2.4 | 83.4 | 3.6 | 90.1 | 0.0 | 85.1 | 2.8 | 81.1 | In |
|  | Development | 2.0 | 88.8 | 2.1 | 90.1 | 0.0 | 86.5 | 1.9 | 84.9 | In |
| 17: Anticipated short-term disadvantages (including complications) occurring after the innovative procedure | Early | 2.9 | 79.9 | 3.6 | 84.2 | 1.4 | 79.7 | 4.7 | 72.6 | In |
|  | Development | 1.5 | 83.8 | 0.7 | 87.1 | 0.7 | 80.4 | 3.8 | 73.6 | In |
| 18: Unanticipated short-term disadvantages (including complications) occurring after the innovative procedure | Early | 1.0 | 81.9 | 3.6 | 83.5 | 0.0 | 83.1 | 2.8 | 75.5 | In |
|  | Development | 1.0 | 87.3 | 0.7 | 84.9 | 0.0 | 83.8 | 1.9 | 78.3 | In |
| 19: Anticipated long-term disadvantages (including complications) after the innovative procedure | Early | 9.8 | 64.7 | 3.7 | 85.4 | 2.0 | 52.0 | 2.8 | 69.8 | No consensus^d^ |
|  | Development | 6.9 | 70.6 | 1.5 | 86.1 | 1.4 | 67.6 | 0.9 | 75.5 | No consensus^c^ |
| 20: Unanticipated long-term disadvantages (including complications) after the innovative procedure | Early | 8.8 | 65.2 | 5.8 | 84.7 | 2.0 | 50.7 | 3.8 | 70.8 | No consensus^c^ |
|  | Development | 6.4 | 71.6 | 2.9 | 84.7 | 1.4 | 67.6 | 2.8 | 76.4 | No consensus^c^ |
| 21: Whether fewer or more resources were required before the innovative procedure, specifically because of the innovative procedure (e.g. fewer blood tests or more scans/pre-operative appointments) | Early | 12.3 | 55.7 | 11.0 | 65.4 | 5.4 | 25.7 | 3.8 | 32.1 | No consensus^e^ |
|  | Development | 7.9 | 64.0 | 5.9 | 69.9 | 4.1 | 37.8 | 1.9 | 41.5 | No consensus^e^ |
| 22: Whether fewer or more resources were required during the innovative procedure, specifically because of the innovative procedure  (e.g. fewer staff, more equipment) | Early | 11.3 | 56.2 | 9.6 | 71.3 | 6.1 | 33.8 | 5.7 | 45.3 | No consensus^e^ |
|  | Development | 6.4 | 64.5 | 2.9 | 79.4 | 4.1 | 40.5 | 2.8 | 50.0 | No consensus^d^ |
| 23: How long the innovative procedure took | Early | 11.8 | 57.6 | 9.7 | 65.7 | 2.7 | 28.4 | 8.5 | 33.0 | No consensus^e^ |
|  | Development | 6.4 | 66.5 | 6.0 | 72.4 | 2.0 | 41.2 | 6.6 | 38.7 | No consensus^e^ |
| 24: Whether fewer or more resources were required during the post-operative hospital stay, specifically because of the innovative procedure (e.g. less/more time in intensive care unit/high dependency unit) | Early | 14.3 | 57.6 | 9.7 | 71.6 | 5.4 | 31.8 | 3.8 | 48.1 | No consensus^e^ |
|  | Development | 7.4 | 65.0 | 5.2 | 76.1 | 2.0 | 41.9 | 0.9 | 50.9 | No consensus^d^ |
| 25: How long the patient(s) stayed in hospital | Early | 16.8 | 50.7 | 9.8 | 66.2 | 5.4 | 25.7 | 6.6 | 38.7 | No consensus^e^ |
|  | Development | 10.3 | 56.2 | 5.3 | 73.7 | 2.7 | 34.5 | 5.7 | 49.1 | No consensus^e^ |
| 26: Whether fewer or more resources were required after leaving hospital, specifically because of the innovative procedure (e.g. fewer out-patient department visits, more tests/scans) | Early | 16.3 | 45.3 | 9.8 | 67.7 | 5.4 | 22.3 | 7.6 | 40.6 | No consensus^e^ |
|  | Development | 8.9 | 55.2 | 4.5 | 72.2 | 3.4 | 29.7 | 2.8 | 53.8 | No consensus^d^ |
| 27: Patients' physical experiences during the innovative procedure (e.g. ability to lie still for duration of procedure) | Early | 10.8 | 57.6 | 3.0 | 82.7 | 4.1 | 46.0 | 1.9 | 63.8 | No consensus^d^ |
|  | Development | 7.4 | 65.0 | 1.5 | 82.7 | 2.0 | 50.7 | 1.0 | 73.3 | No consensus^c^ |
| 28: Patients' psychological or emotional experiences of the innovative procedure (e.g. anxiety associated with having an innovative procedure) | Early | 9.9 | 53.7 | 6.0 | 79.0 | 4.7 | 41.9 | 2.9 | 71.4 | No consensus^c^ |
|  | Development | 7.9 | 58.1 | 4.5 | 82.0 | 2.7 | 48.7 | 1.0 | 74.3 | No consensus^c^ |
| 29: Patient's experiences following the innovative procedure (e.g. pain, physical function) | Early | 4.4 | 70.4 | 2.3 | 90.2 | 1.4 | 61.5 | 1.0 | 81.0 | No consensus^c^ |
|  | Development | 3.0 | 78.8 | 1.5 | 92.5 | 1.4 | 66.2 | 0.0 | 84.8 | No consensus^c^ |
| 30: Whether changes were made to which patients were offered the innovative procedure during the course of the study (e.g. procedure only offered to patients with fewer health problems) | Early | 6.4 | 65.5 | 8.3 | 73.7 | 0.7 | 67.6 | 2.9 | 72.4 | No consensus^c^ |
|  | Development | 3.0 | 76.9 | 3.8 | 79.0 | 0.7 | 72.3 | 1.0 | 75.2 | In |
| 31: Details of which future patients would be considered suitable or not for the innovative procedure | Early | 9.4 | 60.1 | 9.9 | 67.2 | 3.4 | 39.9 | 5.7 | 48.6 | No consensus^e^ |
|  | Development | 5.9 | 69.0 | 6.9 | 70.2 | 2.0 | 45.3 | 2.9 | 52.4 | No consensus^d^ |
| 32: Details of operator(s')* training or expertise that would be considered necessary to perform the innovative procedure in the future  **this term applies to anyone performing the procedure* | Early | 9.4 | 63.1 | 4.6 | 80.2 | 4.1 | 49.3 | 1.0 | 61.0 | No consensus^d^ |
|  | Development | 5.4 | 72.9 | 2.3 | 87.0 | 2.0 | 67.6 | 0.0 | 74.3 | No consensus^c^ |

Gray shading represents items scored as very important (8–9) by ≥70% of participants in the respective stakeholder group

^a^ includes participants who completed at least 1 survey item

^b^ Domain further sub-categorized: ^c^ Scored ‘very important’ (median 8-9) by ≥70% of either patients *or* professionals; ^d^ Scored ‘very important’ (median 8-9) by 50-70% of either patients *or* professionals; ^e^ Scored ‘very important’ (median 8-9) by ≤50% of both patients *and* professionals
